# On Poisons in Relation to Medical Jurisprudence and Medicine

**Published:** 1848-02

**Authors:** 


### BIBLIOGRAPHICAL NOTICES.

*On Poisons in relation to Medical Jurisprudence and Medicine.* By Alfred S. Taylor, F. R. S., Lecturer on Medical Jurisprudence and Chemistry in Guy's Hospital, and Author of "Medical Jurisprudence." *Edited, with notes and additions, by* R. Eglesfeld Griffith, M. D., &c. 8vo. pp. 687. Lea & Blanchard. Philadelphia: 1848.

Mr. Taylor's former work, (on Medical Jurisprudence) has been so favourably received as to induce him to come before the public again, in a more extended view of *toxicology* than he was able to introduce into that treatise. "The crime of poisoning," he remarks, "has been of late so fearfully on the increase, that it seems essential for the proper administration of justice, and for the security of society, to collect and arrange in a convenient form for reference, those important medical facts in relation to death from poison, which, while they constitute a safe guide to the barrister and medical practitioner, may prevent the condemnation of the innocent, and secure the conviction of the guilty." Not only are such treatises necessary for the physician and jurist, but, such is the rapid progress of the various collateral sciences, and chemistry in particular, that a single year brings forth knowledge sufficiently important to demand a new work, or the most complete revision of those already published. For the preparation of such a work, Mr. Taylor is eminently fitted, as every one must admit who has read his "Medical Jurisprudence."

We are glad to observe that Mr. Taylor continues to discard the absurd division of poisons into "animal, vegetable, and mineral," attempted to be revived by M. Flandin. He recognises the three classes of the generality of modern writers on this subject—viz.: "*Irritants*," "*Narcotics*," "Narcotico-Irritants," or "Acro-Narcotics" of some authors.

The author, we perceive, includes in the list of poisons various substances which we are little accustomed to regard as belonging to that class; as the Sulphate of Magnesia, Chloride of Sodium, &c. Well authenticated cases of death caused by these articles, taken in improper quantity, are cited, which seem to justify him in thus regarding them. His doctrine is, that "in Medical Jurisprudence it is necessary to look to the noxious effects produced by particular substances on the system, and the adequacy of these substances to cause death under sysmptoms of poisoning, rather than to the mere quantities in which they may have been taken." It appears to have been decided in the English courts of law, according to Mr. Taylor, that the term "*deadly*," as applied to poisons, is not essential to the validity of an indictment—that it is mere surplusage, and used in legal proceedings only, in pursuance of an ancient form, without any legal consequence. The Judge, (Erle,) in the case cited by the author, laid it down as the law, that "It would be sufficient to describe it (the article taken or administered) simply as a poison, and under that term would fall anything calculated to destroy life. Substances harmless in themselves might become poisons by the time or manner of their administration." This is certainly a most comprehensive rule, according to which any physician may become subject to indictment, in consequence of some idiosyncrasy of a patient of which he could know nothing; or the cook in the kitchen or the lady of the house, because "the lord of the household," or "stranger guest," may chance to die from dining on lobster salad or an oyster pie!

The volume before us comprises a full consideration of all questions of a practical bearing on toxicology, illustrated by numerous cases of great interest, many of which have not before been published. In the latter respect, it is particularly rich—indeed, throughout, the volume is replete with the most valuable information on this important branch of jurisprudence.

